# Malaria prevalence in Bata district, Equatorial Guinea: a cross-sectional study

**DOI:** 10.1186/s12936-015-0986-7

**Published:** 2015-11-16

**Authors:** Policarpo Ncogo, Zaida Herrador, Maria Romay-Barja, Emely García-Carrasco, Gloria Nseng, Pedro Berzosa, Maria A. Santana-Morales, Matilde Riloha, Pilar Aparicio, Basilio Valladares, Agustín Benito

**Affiliations:** Reference Centre for Epidemics Control of Equatorial Guinea (CRCE), Ministry of Health and Social Welfare, Malabo, Equatorial Guinea; National Centre of Tropical Medicine, Instituto de Salud Carlos III (ISCIII) C/Sinesio Delgado, 6-Pabellón 13, 28029 Madrid, Spain; The Spanish Tropical Diseases Research Network (RICET in Spanish), Madrid, Spain; Department of Preventive Medicine, University Hospital of Albacete, Albacete, Spain; Ministry of Health and Social Welfare, Malabo, Equatorial Guinea; Instituto Universitario de Enfermedades Tropicales y Salud Pública de Canarias, Universidad de la Laguna, La Laguna, Canary Islands Spain

**Keywords:** Malaria, Prevalence, Rapid diagnosis test, Anaemia, Equatorial Guinea

## Abstract

**Background:**

Malaria has traditionally been a leading public health problem in Equatorial Guinea. After completion, in September 2011, of the integrated set of interventions against malaria launched by the Global Fund Malaria Programme in the mainland area, the epidemiological situation of malaria remains unknown. The aim of this study was to investigate the prevalence rate of malaria and associated factors based on the rapid diagnosis test (RDT) in Bata district, in order to provide evidence that will reinforce the National Malaria Control Programme.

**Methods:**

From June to August 2013, a representative cross sectional survey using a multistage, stratified, cluster-selected sample was carried out in urban zones and rural villages from Bata district. Data on socio-demographic, health status and malaria-related behaviours was collected. Malaria diagnosis was performed by RDT. Bivariate and multivariable statistical methods were employed to assess malaria prevalence and its association with different factors.

**Results:**

Prevalence of malaria was higher in rural settings (58.9 %; CI 95 % 55.2–62.5 %) than in the sampled urban communities (33.9 %; CI 95 % 31.1–36.9 %). Presence of anaemia was also high, especially in rural sites (89.6 vs. 82.8 %, p < 0.001). The analyses show that a positive RDT result was significantly associated with age group, the most affected age range being 13 months–14 years old. Other significant covariates were ethnic group (only in urban sites), number of adults living in the house (only in rural villages) previous history of fever, anaemia (only in urban sites) and sleeping under a bed net. Moreover, those who never slept under a bed net were two times more likely to have malaria.

**Conclusion:**

The prevalence of malaria was high in Bata district, especially in rural villages. The National Programme to fight malaria in Equatorial Guinea should take into account the differences found between rural and urban communities and age groups to target appropriately those worst affected. The findings of this study will assist in planning and undertaking regional policy and other preventive initiatives.

**Electronic supplementary material:**

The online version of this article (doi:10.1186/s12936-015-0986-7) contains supplementary material, which is available to authorized users.

## Background

Equatorial Guinea (EG) consists of two parts, a mainland and an insular region (Bioko as the main island). In both regions, malaria is an holo-endemic disease and exhibits a year-round transmission pattern [1, 2]. In 2014, up to 13,000 malaria cases and 66 deaths were reported [3]. Overall, it account for 21 % of the causes of death among children under five years of age in 2010 [4].

Four out of five species of *Plasmodium* responsible for malaria disease in humans can be found in EG. Particularly in the mainland, the following distribution was described in 2011, 95.2 % for *Plasmodium falciparum* and *Plasmodium vivax* 9.5 %, with eight cases of mixed infection [5]. The tropical all year round humid climate and the presence of several rivers and streams, both fast and slow flowing, provides ideal breeding conditions for different malaria vectors [6, 7]. Entomological studies have shown *Anopheles gambiae* sensu lato (s.l.) and *Anopheles funestus* to be the major vectors of malaria, though the presence of other species, such as *Anopheles melas* and *Anopheles moucheti* have also been reported [6, 8].

The current malaria burden differ between the mainland and the island, being considerably lower in the Bioko island than in the continent [9, 10]. This is mainly due to The Bioko Island Malaria Control Project (BIMCP), which was launched in 2004 using a wide range of control measures with the aim of substantially reducing, and ultimately eliminating malaria from the island [10, 11]. After 10 years of its implementation, malaria transmission had decreased by nearly 70 percent among children 2–14 years old, and malaria-related deaths in children under 5 years of age had declined by 65 % [12].

Following the success of the BIMCP [9], this project was extended to the mainland area under the Equatorial Guinea Malaria Control Initiative (EGMCI) in 2007 [7]. Vector control formed the basis of the initiative and consisted of indoor residual spraying (IRS). An evaluation of the impact of this initiative was carried out 4 years after its implementation, showing a decrease in the estimated prevalence of *P. falciparum* in children between 1 and 4 years of age, from 68 % in 2007 to 52 % in 2011 [13]. Besides, data from the 2011 Health Survey (EDSGE-I) showed RDT-based malaria prevalence of 58.7 % in children aged 6–59 months old living in the mainland [14]. Unfortunately, the EGMCI was suspended in 2011 due to funding restrictions [11]. Since then, no prevalence data has been reported from the mainland. The present study aimed to describe the current prevalence of malaria, and their related factors, in the District of Bata, in Equatorial Guinea.

## Methods

### Study area and population

The EG mainland geographical region covers 26,017 km^2^ and it is bordered by Cameroon on the north and Gabon on the south and east. The mainland region currently has a population of about 315,625, mainly composed of ethnic Fang tribes [15]. The proportion of population living in urban areas has increased from 27.1 % in 1975 to 48.3 % in 2003, partly due to the industrial development experienced by this country since mid-’90 s [16]. The mainland region comprises four provinces: Centro Sur, Kie-Ntem, Litoral and Wele-Nzas. In turn, each province is divided into several districts. Litoral province has the largest population of EG and its capital is Bata. According to the last available census (2001), the population in the Bata district is 230,283 inhabitants, of which 115,077 are men (49.50 %) and 115,206 women (50.50 %) [17]. Bata has a tropical climate with two dry seasons (December to March and from June to September) alternating with two rainy seasons (March to June and September to December). Mean daily maximum and minimum temperatures range between 29–32 and 19–22 °C, respectively.

### Study design

This cross–sectional study was carried out during June–August 2013 in the Bata district. It was part of a project called “Prevamal”, which aimed to provide baseline data on malaria prevalence, molecular characterization of *Plasmodium* and malaria vectors in the area and information on knowledge, practices and attitudes among the targeted population.

Sampling was carried out with the use of a multistage, stratified cluster strategy. The strata were rural and urban settings, using an expected malaria prevalence of 50 %. The initial sample size was increased in prevision of missing data but replacement was not carried out at any of the sampling stages and included 1762 individuals and 440 households

First, rural villages and urban neighbourhoods were randomly selected with probability proportional to size to improve accuracy in sample design. Second sampling units were randomly selected households from an updated census from each cluster provided by the head of the village or neighbourhood. All children with reported age between 2 months and 14 years living in the selected household and not receiving malaria treatment at the moment of the survey were included in the study. Only one adult 15 years of age and older per household was selected randomly, from a list with all the adults. A total of 1043 and 698 people living in urban and rural settings, respectively, were recruited.

### Data collection

A closed ended pre-piloted questionnaire was administered to the household caretaker/head of the participant children and to the randomly selected adult by trained medical personnel (nurses). The questionnaire comprised the following parts: demographic characteristics, health status and history of previous episodes of malaria, and malaria-related behaviours. The questionnaire was previously translated into the main local language, Fang, and the option was given to the care provider to be interviewed in Spanish or Fang, which is one of the official languages in the country.

Haemoglobin (Hb) levels for children and pregnant women were ascertained using HemoCue (Ängelholm, Sweden). NADAL rapid diagnostic test (Nal von Minden, Moers, Germany) for malaria infection was performed in situ. Rapid diagnostic tests (RDTs) assist in the diagnosis of malaria by detecting evidence of malaria parasites in human blood and are an alternative to diagnosis based on clinical grounds or microscopy. The test is capable of detecting both *P. falciparum* and other *Plasmodium* species. Participants with positive rapid tests were immediately offered treatment according to the National Guidelines. The anaemic children and pregnant women received iron supplementation. Anaemia was defined according to WHO criteria [18].

### Statistical analysis

The collected data were double entered into a data entry file using EpiData software, V.3.1. Data were then transferred to SPSS version 18.0 (SPSS Inc., Chicago, Illinois, USA) for data management and analysed according to the study objectives. The prevalence rates by cluster and setting were mapped using the Geographical Information System Arcgis version 10.0.

The outcome of interest was malaria RDT result. The response variable was binary, indicating whether or not a person was positive for malaria. The independent covariates comprised individual variables, that included sex, age, ethnic group, literacy, family source of income, number of children and number of adults, fever in the last week/last 24 h, current physical discomfort, drug intake, episode of malaria in the last year, sleeping outside the house, use of bed net, and use of spray or mosquito coil. Family source of income, number of children and number of adults were the only variables collected at household level.

Frequencies and percentages were used to summarize data and to explore the differences by socio-demographic variables. To assess factors related to malaria prevalence, Student’s *t* test and *χ*^2^ tests for continuous and categorical variables, respectively, were performed, with stratification by setting. Comparisons for which p values were below 0.05 were considered significant. Age and sex, considered biologically relevant, and all variables associated with each of the outcomes at the p < 0.10 level were included in the multivariable analysis. Logistic regression models stratified by setting were obtained by using a manual backward stepwise procedure. The major assumptions of logistic regression analysis (absence of multicollinearity and interaction among independent variables) were checked to be satisfied. The goodness of fit was assessed by using Hosmer–Lemeshow statistic. The adjusted odds ratio (aOR) and 95 % confidence interval (95 % CI) were computed. P values less than or equal to 0.05 were considered statistically significant.

### Ethical clearance

The study was approved by the ethical review board of the Health Institute Carlos III (ISCIII in Spanish) and the Minister of Health and Social Welfare of Equatorial Guinea (MINSABS). Support letters were obtained from the MINSABS and the Hospital of Bata. The village and neighbourhood representatives were informed by an official letter from the MINSABS of the day of the visit and the scope of the study. Written informed consent was obtained from all patients prior to study inclusion. Consent to publish from the participant (or legal parent or guardian for children) to report the data were also obtained. Anonymity was assured. A written statement was also included on the introductory part of the questionnaires in which further information concerning the purpose of the study and the confidentiality of the research information was given. Data were analysed in anonymous form.

## Results

A total of 26 urban neighbourhoods and 19 rural villages were sampled in Bata district. Sampled clusters contained 272 and 172 households in urban and rural Bata district, respectively, and none declined participation (mean number of households per cluster in both settings: 12). This resulted in a study population of 1043 (mean number of population per household: 3.2 range: 1–16) and 698 persons (mean number of population per household: 3.2 range: 1–13) in urban and rural settings, respectively. Seven children were taking malaria treatment at the time of the study, being therefore excluded from the analysis. A further seven were excluded for various reasons. RDT was finally performed in 1727 out of 1741 individuals (99.2 %).

### Characteristics of study population

Individual characteristics are summarized in Table 1, stratified by setting. In urban areas, 56.1 % were females and the predominant age group was 13 months–5 years (30.7 %), whereas the predominant age-group in rural sites was 6–14 years (31.8 %, p < 0.001). Those living in urban areas knew how to read and write more frequently than rural ones (71.6 vs. 59.8 % respectively, p < 0.001). The proportion of households with more than three children and five or more adults permanently living in the house was higher in rural sites (p < 0.005).

With regards to the interviewees´ health status, 7.2 and 2.9 % from rural and urban settings, respectively, had fever at the moment of the survey (p < 0.001). The prevalence of anaemia was up to 80 % in both settings, slightly higher in rural sites (p < 0.001). Whereas 73.1 % of participants from urban communities said that they sleep always under a bed net, only 36.7 % of those living in rural areas referred this behaviour (p < 0.001).

### *Plasmodium* prevalence and associated factors in urban and rural settings

**Fig. 1:**
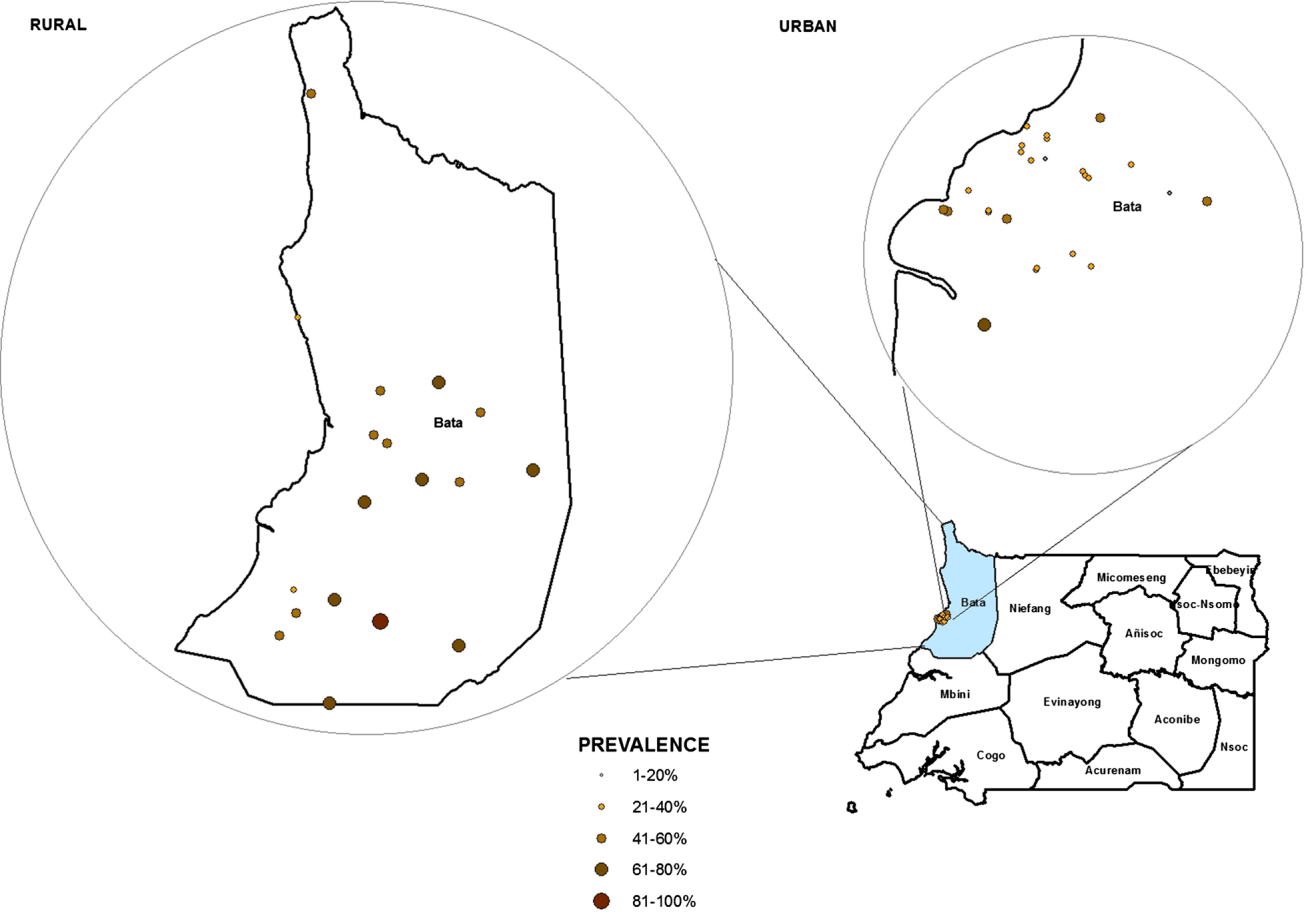
Distribution of malaria prevalence in rural and urban communities of the District of Bata, Equatorial Guinea, June–August 2013

Overall RDT-based prevalence of malaria was 46.2 % in Bata district. Prevalence of malaria was higher in most rural clusters than in the sampled urban communities. The range varied from 18.4 to 62.8 % in urban sites to 30.8–89.7 % in rural places (Fig. 1).

**Fig. 2:**
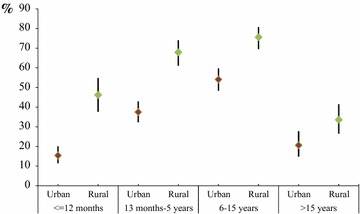
Prevalence of Plasmodium infection (and its 95 % CI) by age group and setting, Bata district, Equatorial Guinea, June–August 2013

Mean prevalence was 33.9 % (95 % CI 31.1–36.9) and 58.9 % (95 % CI 55.2–62.5) in urban and rural settings, respectively (p < 0.001). Overall, RDT based malaria prevalences were higher in rural sites for all age groups (Additional file 1). Prevalence in children aged less or equal than 12 months was 15.4 % (95 % CI 11.4–20.1) and 46.2 % (95 % CI 37.7–54.8) in urban and rural settings, respectively (p < 0.001, Fig. 2).

The prevalence of malaria was similar in males and females in urban settings, but not in rural sites, where a higher percentage of males were RDT positive (p = 0.002). Prevalence was higher in age groups 13 months–5 years and 6–14 years old in both settings (p < 0.001). In urban communities, the interviewees from the Combe ethnic group were less affected by malaria (22.4 %) than the Fang ethnic group (34.7 %, p = 0.053). In rural sites, malaria RDT-based prevalence was higher in those households with a greater number of adults (p = 0.006). Regarding participants´ health status, fever in the last 24 h (p < 0.001), report of any kind of discomfort (p = 0.004) and having anaemia (p < 0.001) were significantly malaria-associated factors in urban settings, while in rural sites only current fever shown association with the presence of malaria (p = 0.011). With respect to malaria related-behaviours, prevalence of malaria was significantly lower in those participants who reported to sleep always under a bed net than those who referred that they never sleep under a bed net (28 vs. 54 % and 48.6 vs. 68.2 % in urban and rural settings, respectively, p < 0.001, Table 2).

**Table 1:** Individual characteristics, health status and malaria-related behaviours, Bata district, Equatorial Guinea, June–August 2013

Characteristics	Urban (n = 1043)		Rural (n = 698)		p value
	No.	(%)	No.	(%)	
*Individual characteristics*					
Sex					
Male	458	43.91	297	42.55	0.574
Female	585	56.09	401	57.45	
Age group					
≤12 months	273	26.17	130	18.62	<0.001
13 months–5 years	320	30.68	197	28.22	
6–15 years	294	28.19	222	31.81	
≥15 years	156	14.96	149	21.35	
Ethnic group					
Fang	849	81.40	600	85.96	0.040
Combe	87	8.34	41	5.87	
Other	107	10.26	57	8.17	
Know how to read and write^a^					
Yes	322	71.56	222	59.84	0.002
No	121	26.89	141	38.01	
Don’t know/Don’t answer	7	1.56	8	2.16	
*Household characteristics*					
Family source of income					
Agriculture, forestry, fishing and hunting sector	11	1.10	154	23.23	<0.001
Employment as employee	597	59.88	251	37.86	
Self-employed	311	31.19	212	31.98	
Other	78	7.82	46	6.94	
Number of children					
≤3 children	348	33.37	201	28.80	0.044
>3 children	695	66.63	497	71.20	
Number of adults					
≤2 adults	235	22.53	151	21.63	0.007
3–4 adults	420	40.27	237	33.95	
≥5 adults	388	37.20	310	44.41	
*Health status*					
Did you have had fever in the last 24 h?					
Yes	84	8.15	81	11.76	0.029
No	945	91.66	605	87.81	
Don’t know/Don’t answer	2	0.19	3	0.44	
Do you have any discomfort at present time?					
Yes	100	9.83	144	21.05	<0.001
No	915	89.97	538	78.65	
Don’t know/Don’t answer	2	0.20	2	0.29	
Are you taking any drug at the present time?					
Yes	105	10.49	117	17.11	<0.001
No	894	89.31	567	82.89	
Don’t know/Don’t answer	2	0.20	0	0.00	
Did you have malaria the last year?					
Yes	514	49.52	365	52.90	0.168
No	515	49.61	315	45.65	
Don’t know/Don’t answer	9	0.87	10	1.45	
Current fever					
Yes	30	2.91	50	7.16	<0.001
No	1002	97.09	648	92.84	
Anaemia^b^					
Yes	749	82.85	499	89.59	<0.001
No	155	17.15	58	10.41	
*Malaria related-behaviours*					
Do you sleep outside the house?					
Always	13	1.26	17	2.47	0.024
Sometimes	16	1.55	18	2.61	
Never	1002	97.09	650	94.34	
Don’t know/Don’t answer	1	0.10	4	0.58	
Do you sleep under a bed net?					
Always	754	73.06	253	36.67	<0.001
Sometimes	50	4.84	69	10.00	
Never	227	22.00	365	52.90	
Don’t know/Don’t answer	1	0.10	3	0.43	
Do you use spray or mosquito coil?					
Always	21	2.04	6	0.87	0.185
Sometimes	18	1.75	15	2.17	
Never	990	96.02	666	96.52	
Don’t know/Don’t answer	2	0.19	3	0.43	

^a^Only ≥6 years old

^b^Only <15 years old

**Table 2:** Factors associated with *Plasmodium* infection in Bata district, Equatorial Guinea, June–August 2013

Characteristics	Urban (n = 1031)			Rural (n = 696)		
	No.	(%)	p value	No.	(%)	p value
*Individual characteristics*						
Sex						
Male	165	36.59	0.115	194	65.54	0.002
Female	185	31.90		216	54.00	
Age group						
≤12 months	41	15.36	<0.001	60	46.15	<0.001
13 months–5 years	119	37.54		133	67.86	
6–15 years	158	54.11		167	75.57	
≥15 years	32	20.65		50	33.56	
Ethnic group						
Fang	292	34.68	0.053	361	60.27	0.168
Combe	19	22.35		22	53.66	
Other	39	37.50		27	48.21	
*Household characteristics*						
Family source of income						
Agriculture, forestry, fishing and hunting sector	6	54.55	0.309	95	62.09	0.373
Employment as employee	198	33.45		155	62.00	
Self-employed	115	37.58		116	54.72	
Other	25	32.47		28	60.87	
Number of children						
≤3 children	113	32.94	0.631	111	55.22	0.208
>3 children	237	34.45		299	60.40	
Number of adults						
≤12 adults	92	39.66	0.113	72	47.68	0.006
3–4 adults	133	32.13		144	61.28	
≥5 adults	125	32.47		194	62.58	
*Health status*						
Did you have fever in the last 24 h?						
No	298	31.87	<0.001	343	56.79	0.007
Yes	45	54.88		58	72.50	
Do you have any discomfort at present time						
No	293	32.38	0.004	308	57.46	0.090
Yes	46	46.94		94	65.28	
Did you have malaria the last year?						
No	158	31.04	0.060	178	56.51	0.280
Yes	186	36.61		220	60.61	
Current fever						
No	336	33.91	0.753	372	57.59	0.011
Yes	11	36.67		38	76.00	
Anaemia						
No	32	20.92	<0.001	34	59.65	0.351
Yes	290	39.14		328	65.86	
*Malaria related-behaviours*						
Do you sleep outside the house?						
Always	2	15.38	0.071	8	47.06	0.265
Sometimes	2	12.50		8	44.44	
Never	339	34.24		386	59.57	
Do you sleep under a bed net?						
Always	209	28.05	<0.001	122	48.61	<0.001
Sometimes	13	26.00		32	46.38	
Never	121	54.02		249	68.22	
Do you use spray or mosquito coil?						
Always	10	47.62	0.051	3	50.00	0.469
Sometimes	10	55.56		11	73.33	
Never	322	32.92		389	58.58	

Results from the multivariable logistic analysis are summarized in Table 3. Sex difference in rural prevalence rate did not persist after adjustment was carried out, while same significant age group differences remained in the model for both type of settings. Those who belonged to the Combe ethnic group were almost two times less likely to have malaria (95 % CI 0.26–0.90) than study participants from the ethnic group Fang in urban communities. Interviewees living in a house with five or more adults were 1.74 times more likely to have malaria (95 % CI 1.10–2.74) than those living in in rural households with two or less adults. Having fever in the last 24 h was positively associated to the current presence of malaria in both types of setting (p < 0.005), while anaemia and malaria were only significantly associated in urban sites (OR 3.02; 95 % CI 1.92–4.76). Study participants who referred that they never sleep under a bed net were 2.2 and 1.7 times more likely to have malaria that those who said that they always sleep under a bed net in urban and rural communities, respectively.

**Table 3:** Multivariate logistic regression analysis of Plasmodium infection stratified by setting, Bata district, Equatorial Guinea, June–August 2013

Characteristics	Urban (n = 1031)			Rural (n = 696)		
	aOR	95 % CI	p value	aOR	95 % CI	p value
*Individual characteristics*						
Sex						
Male	NS	NS	NS	NS	NS	NS
Female						
Age group						
≤12 months	1			1		
13 months–5 years	3.57	(2.31–5.52)	<0.001	2.44	(1.48–4.01)	<0.001
6–15 years	7.65	(4.89–11.97)	<0.001	3.58	(2.16–5.93)	<0.001
≥15 years	1.69	(0.62–4.59)	0.304	0.56	(0.33–0.95)	0.031
Ethnic group						
Fang	1			NS	NS	NS
Combe	0.48	(0.26–0.90)	0.021			
Other	0.93	(0.55–1.56)	0.779			
*Household characteristics*						
Number of adults						
≤2 adults	NS	NS	NS	1		
3–4 adults				1.56	(0.98–2.48)	0.062
≥5 adults				1.74	(1.10–2.74)	0.017
*Health status*						
Did you have fever in the last 24 h?						
No	1		0.001	1		
Yes	2.57	(1.45–4.55)		2.32	(1.32–4.08)	0.004
Anemia						
No	1		<0.001	NS	NS	NS
Yes	3.02	(1.92–4.76)				
*Malaria related-behaviours*						
Do you sleep under a bed net?						
Always	1			1		
Sometimes	0.56	(0.26–1.20)	0.135	0.80	(0.45–1.45)	0.468
Never	2.20	(1.52–3.18)	0.000	1.77	(1.23–2.57)	0.002
Hosmer–Lemeshow test	p = 0.623			p = 0.215		

## Discussion

This study documented a high prevalence of malaria in Bata district, in mainland Equatorial Guinea. This is consistent with results from previous years, when the estimates of community prevalence of infection exceeded 50 % [13, 16]. While a declining prevalence of malaria, attributed to interventions to prevent and/or treat malaria, have been reported in Bioko Island [10], continental Equatorial Guinea still seem to be exposed to one of the highest levels of malaria infection in the world [19]. Malaria prevalence was significantly higher in rural communities, and various factors were associated with higher prevalence of malaria in one or both settings. From previuos knowledge, this is the first representative survey for both urban and rural settings carried out in Bata district. Thus, these results will assist stakeholders in planning and undertaking evidence-based policy initiatives.

The overall prevalence of malaria determined by RDTs was 46.2 % in Bata district. This is consistent with results from the WHO malaria report 2014 [3]. The malaria prevalence in rural villages was 25 % above urban settings, which stresses the overwhelming burden of malaria in rural areas. These regional differences have been previously observed in EG [14, 16] and other developing countries [20, 21]. Many reasons for these regional disparities in sub-Saharan Africa have been given in the literature; among others: the higher numbers and levels of vector breeding sites in rural areas [22] and the better physical access to health services and a greater use of insecticide-treated nets (ITN) in urban settings [23, 24].

People living in rural villages referred worst health status and lower adherence to malaria-related prevention activities, which might, at least partially, explain the differences founded. Rural–urban prevalence disparities were found for all age groups, though bigger differences were observed for children less than 5 years of age (prevalence was up to 30 % higher in rural villages). In a nationally representative survey of children 0–5 years old carried out in EG, overall prevalence was also higher in rural (58.8 %) compared to urban areas (44.0 %) [16], though the difference was not as noteworthy as ours. Bigger urban–rural disparities were reported in children aged 6–59 months old by the 2011 National Health Survey EDSGE-I (30 vs 62.6 %, respectively) [14], which is more in line with our results. These age group differences by setting might be related to differences on malaria related behaviours and socio-demographic determinants [23, 25], although further assessment is needed to prove this hypothesis in this particular district.

Study participants aged 13 months–5 years and 6–14 years old were the most affected by malaria in both settings. This result is in line with earlier reports highlighting that children bear the greatest burden of malaria [1, 21]. Even so, the high prevalence observed in the age group 13 months–5 years is striking, considering that this age group is usually reckoned as highly vulnerable to malaria, and therefore most frequently targeted group, together with pregnant women, by prevention and control interventions [13, 23, 26]. The lack of programmed campaigns after the end of EGMCI in September 2011 may explain these poor results. Regarding the 6–15 years old age group, it constitutes the group with significantly higher risk of malaria in our study population. This age group fall within those with low partial immunity in an endemic area [27]. Moreover, malaria risk is increased in school-aged children because they usually spend more time outside [28], especially during school holidays. This could be a possible explanation since our research mostly coincided with school summer holidays in the country.

Children aged less than 1 year old and adults constituted the less affected age groups in both settings. During the first months of life, the risk of infection is lower because there is still a degree of immunity from the mother [16]. Moreover, the use of bed nets and other preventive activities have been shown to be more frequent in children under 1 year of age in Equatorial Guinea [14, 16]. Even if malaria prevalence was lower in this age group, there was a remarkable difference in malaria prevalence rates by setting (15.4 vs 46.2 % in urban and rural areas, respectively). This huge disparity might be due to less common use of bed nets in rural sites, as it was shown in the EDSGE-I [14], environmental [29] and nutritional factors [16] and/or socio-demographic determinants [25], among others. With regards to adult population, it is known that the risk of infection first increases with age and starts decreasing when the individual himself reaches a degree of immunity due to repeated exposure to the parasite [30], especially in holo-endemic areas [31]. This might explain the lower prevalence among adult populations found in both settings.

In urban neighbourhoods, the prevalence of malaria was two times higher in the Fang ethnic group, the largest in the country, than in the Combe, a coastal ethnic group. Difference in susceptibility to malaria between ethnic groups has been previously described in other sub-Saharan countries, even in spite of similar sociocultural factors and entomologic inoculation rates [32, 33]. In Equatorial Guinea, Roche et al. attributed the higher prevalence of malaria parasites, in the continental region (Fang ethnic population) than on the Bioko Island (mainly Bubi ethnic population) to ethnic differences and human movements [8]. Moreover, environmental and cultural factors may impact malaria prevalence in this context, and therefore further investigated on next studies.

Interviewees from rural villages were almost two times more prompted to be RDT positive if the home was overcrowded (in terms of number of adults living in the house). This result is in line with previous researches showing the importance of housing conditions in malaria indoor transmission [24, 34]. As expected, those study participants who referred to have fever in the 24 h previous to the survey were up to two times more likely to be RDT positive. Overall, there was a high prevalence of anaemia (86 %), greater in rural villages but, interestingly, only significantly associated to malaria in urban neighbourhoods. Anaemia is a virtually obligatory symptom of malaria, and severe anaemia constitutes the most frequent defining symptom of severe malaria [35]. However, anaemia is multi-factorial in origin, and although malaria plays a key etiologic role in endemic countries, it is clear that poor nutritional status, micronutrient deficiencies, intestinal helminths, HIV infection, and haemoglobinopathies make also important additional contributions [26]. Therefore, anaemia in rural villages might be related not only to malaria but also to these other factors, which need to be further investigated in future research.

With regards to malaria related behaviours, most study participants said that they never slept outside the house and the use of spray or mosquito coil was almost anecdotal. Notably, 22 % and up to 50 % of interviewees living in urban neighborhoods and rural villages, respectively, referred to never sleep under a bed net. These poor results are in line with those obtained by Custodio et al. in a national survey carried out in EG [16]. Moreover, never sleeping under a bed net increased around two times the risk of malaria in both type of settings in our survey. It is a fact that bed nets, even if they are not insecticide-treated, confer high protection against parasite infection [23, 36, 37], thus this preventive behavior should be enhanced in the study area. According to the knowledge attitudes and practices survey, which was also part of the Prevamal study, the delays in seeking treatment, the type of malaria therapy received and the cost of treatment are the principal problems found in Bata District and recommendations were made towards providing sufficient supplies of effective anti-malarial drugs and improving malaria treatment skills in households and in both public and private sectors [38].

### Limitations

The present study was conducted in Bata district, thus, the findings may not be generalizable to the whole country. Additionally, the cross-sectional nature of this data does not allow to examine causality in the relationship between malaria prevalence and diverse risk factors. Moreover, malaria is endemic in Bata district with year-round transmission, thus seasonality might have some impact in malaria prevalence and, therefore, further assessed by consecutive measurements.

Rapid diagnostic tests (RDTs) were used to estimate malaria prevalence in the study area and some degree of overestimation of prevalence is likely with a HRP2-recognizing RDT tests. Discrepancies in prevalence estimates generated from microscopy testing and from RDT testing exists, and that there are difficult to reconcile [39]. Furthermore, there is a lack of a gold standard diagnostic test for use in national survey settings, especially in those zones of high malaria prevalence [40]. These calls for the need to undertake further investigations not only to substantiate the data obtained in the present study but also to help local program managers to take decisions in the future.

## Conclusions

This study findings show a high prevalence of RDT-based malaria prevalence in Bata district. Malaria continues to be a significant public health problem in the mainland. While the EGMCI is meeting with success most of the WHO global goals and targets in the insular area [3, 12], the EGMCI activities were abruptly interrupted in 2011 in the continental zone due to funding restrictions. Moreover, the high malaria prevalence found in the continental is posing a challenge for the accomplishment of the successful initiatives in the insular part of the country [11]. Therefore, resuming the control activities in the EG mainland becomes very important.

Children aged 13 months–5 years and 6–14 years old and those living in rural settings bear the greatest burden of malaria in Bata district. Some particular risk factors for malaria in urban neighbourhoods and rural villages were identified. To effectively tackle malaria, the National Programme to Fight Malaria in Equatorial Guinea should orient interventions to the local needs, with special emphasis in those worst affected. Particularly in rural areas, the challenge will be for health workers to build on this knowledge through educational campaigns promoting the importance of preventive measures, like the use of bed nets, when advising households about malaria. This study findings will help policy makers plan and undertake new regional initiatives to streamline recommendations. Policymakers and providers are expected to evaluate resources allocation and act accordingly. The study finding will also assist at improving the preventive practices and the treatment accessibility, as well as extending the study in the rest of the country.

## Additional file

10.1186/s12936-015-0986-7 Prevalence of Plasmodium infection by age group and setting, Bata district, Equatorial Guinea, June – August 2013.

## Abbreviations

aOR: adjusted odds ratio
BIMCP: Bioko Island Malaria Control Project
CRCE: Centro de Referencia de Control de Endemias
EDSGE-I: First National Health Survey in Equatorial Guinea
EG: Equatorial Guinea
EGMCI: Equatorial Guinea Malaria Control Initiative
Hb: hemoglobin
ISCIII: Instituto de Salud Carlos III
MINSABS: Minister of Health and Social Welfare of Equatorial Guinea
NS: Non Significant
OR: odds ratio
RDTs: rapid diagnostic tests
RICET: Network Biomedical Research on Tropical Diseases
WHO: World Health Organization

## References

[1] WHO. World malaria report 2013. Geneva: World Health Organization; 2014. http://www.who.int/malaria/publications/world_malaria_report_2013/en/. Accessed 15 Jan 2015.

[2] Benito A, Roche J, Molina R, Amela C, Alvar J (1994). Application and evaluation of QBC malaria diagnosis in a holoendemic area. Appl Parasitol.

[3] WHO. World malaria report 2014. Geneva: World Health Organization; 2015. http://www.who.int/malaria/publications/world_malaria_report_2014/wmr-2014-no-profiles.pdf. Accessed 15 Jan 2015.

[4] WHO. World Health Statistics 2012. Geneva: World Health Organization; 2013. http://www.who.int/gho/publications/world_health_statistics/EN_WHS2012_Full.pdf. Accessed 17 Jan 2015.

[5] Mendes C, Dias F, Figueiredo J, Mora VG, Cano J, de Sousa B (2011). Duffy negative antigen is no longer a barrier to *Plasmodium vivax*—molecular evidences from the African West Coast (Angola and Equatorial Guinea). PLoS Negl Trop Dis..

[6] Molina R, Benito A, Blanca F, Roche J, Otunga B, Alvar J (1996). The Anophelines of Equatorial Guinea: ethology and susceptibility studies. Res Rev Parasitol..

[7] Ridl FC, Bass C, Torrez M, Govender D, Ramdeen V, Yellot L (2008). A pre-intervention study of malaria vector abundance in Rio Muni, Equatorial Guinea: their role in malaria transmission and the incidence of insecticide resistance alleles. Malar J..

[8] Roche J, Ayecaba S, Amela C, Alvar J, Benito A (1996). Epidemiological characteristics of malaria in Equatorial Guinea. Res Rev Parasitol..

[9] Sharp BL, Ridl FC, Govender D, Kuklinski J, Kleinschmidt I (2007). Malaria vector control by indoor residual insecticide spraying on the tropical island of Bioko, Equatorial Guinea. Malar J..

[10] Overgaard HJ, Reddy VP, Abaga S, Matias A, Reddy MR, Kulkarni V (2012). Malaria transmission after five years of vector control on Bioko Island, Equatorial Guinea. Parasit Vectors..

[11] Bradley J, Monti F, Rehman AM, Schwabe C, Vargas D, Garcia G (2015). Infection importation: a key challenge to malaria elimination on Bioko Island, Equatorial Guinea. Malar J..

[12] The Bioko Island Malaria Control Project (BIMCP). Malaria Control Project. Ten year anniversary. 2014. http://www.marathonoil.com/content/documents/social_responsibility/Bioko_Island_Malaria_Control_Project_Web.pdf. Accessed 5 Feb 2015.

[13] Rehman AM, Mann AG, Schwabe C, Reddy MR, Gomes IR, Slotman MA (2013). Five years of malaria control in the continental region, Equatorial Guinea. Malar J..

[14] Ministerio de Sanidad y Bienestar Social, Ministerio de Economía, Planificación e Inversiones Públicas, ICF International. Encuesta Demográfica y de Salud (EDSGE-I). ICF International Calverton, Maryland, USA; 2012. https://dhsprogram.com/pubs/pdf/FR271/FR271.pdf. Accessed 12 Feb 2015.

[15] Food and Agriculture Organization of the United Nations (FAO). Documento de Perspectiva—República de Guinea Ecuatorial, 2001. http://www.fao.org/docrep/004/ab581s/AB581S00.htm#TOC. Accessed 18 Feb 2015.

[16] Custodio E, Sanchez I, Benito A, Roche J, Descalzo MA, Villamor E (2009). Nutritional and socio-economic factors associated with *Plasmodium falciparum* infection in children from Equatorial Guinea: results from a nationally representative survey. Malar J..

[17] Dirección General de Estadísticas. Censo General de Población y Viviendas del año 2001. Gobierno de la República de Guinea Ecuatoria; 2001.

[18] WHO. Haemoglobin concentrations for the diagnosis of anaemia and assessment of severity. Vitamin and Mineral Nutrition Information System. Geneva: World Health Organization; 2011. _http://www.who.int/vmnis/indicators/haemoglobin.pdf_ Accessed 1 Mar 2015.

[19] Gething PW, Patil AP, Smith DL, Guerra CA, Elyazar I, Johnston GL (2011). A new world malaria map: *Plasmodium falciparum* endemicity in 2010. Malar J..

[20] Pond BS (2013). Malaria indicator surveys demonstrate a markedly lower prevalence of malaria in large cities of sub-Saharan Africa. Malar J..

[21] WHO. World malaria report 2008. Geneva: World Health Organization; 2008. Available: http://www.malaria.org/malaria2008.pdf. Accessed 1 Mar 2015.

[22] Robert V, Macintyre K, Keating J, Trape J-F, Duchemin J-B, Warren M (2003). Malaria transmission in urban sub-Saharan Africa. Am J Trop Med Hyg.

[23] Monasch R, Reinisch A, Steketee RW, Korenromp EL, Alnwick D, Bergevin Y (2004). Child coverage with mosquito nets and malaria treatment from population-based surveys in African countries: a baseline for monitoring progress in roll back malaria. Am J Trop Med Hyg.

[24] Hay SI, Guerra CA, Tatem AJ, Atkinson PM, Snow RW (2005). Tropical infectious diseases: Urbanization, malaria transmission and disease burden in Africa. Nat Rev Microbiol.

[25] Nyarko SH, Cobblah A (2014). Sociodemographic determinants of malaria among under-five children in Ghana. Malar Res Treat..

[26] Crawley J (2004). Reducing the burden of anemia in infants and young children in malaria-endemic countries of Africa: from evidence to action. Am J Trop Med Hyg.

[27] Ndong IC, van Reenen M, Boakye DA, Mbacham WF, Grobler AF (2014). Trends in malaria admissions at the Mbakong Health Centre of the North West Region of Cameroon: a retrospective study. Malar J..

[28] Nankabirwa J, Brooker SJ, Clarke SE, Fernando D, Gitonga CW, Schellenberg D (2014). Malaria in school-age children in Africa: an increasingly important challenge. Trop Med Int Health..

[29] Zoungrana A, Chou Y-J, Pu C (2014). Socioeconomic and environment determinants as predictors of severe malaria in children under 5 years of age admitted in two hospitals in Koudougou district, Burkina Faso: a cross sectional study. Acta Trop.

[30] Carneiro I, Roca-Feltrer A, Griffin JT, Smith L, Tanner M, Schellenberg JA (2010). Age-patterns of malaria vary with severity, transmission intensity and seasonality in sub-Saharan Africa: a systematic review and pooled analysis. PLoS One.

[31] Sharma SK, Chattopadhyay R, Chakrabarti K, Pati SS, Srivastava VK, Tyagi PK (2004). Epidemiology of malaria transmission and development of natural immunity in a malaria-endemic village, San Dulakudar, in Orissa state, India. Am J Trop Med Hyg..

[32] Greenwood B, Groenendaal F, Bradley A, Greenwood A, Shenton F, Tulloch S (1987). Ethnic differences in the prevalence of splenomegaly and malaria in The Gambia. Ann Trop Med Parasitol.

[33] Dolo A, Modiano D, Maiga B, Daou M, Dolo G, Guindo H (2005). Difference in susceptibility to malaria between two sympatric ethnic groups in Mali. Am J Trop Med Hyg.

[34] Koram K, Bennett S, Adiamah J, Greenwood B (1995). Socio-economic risk factors for malaria in a peri-urban area of The Gambia. Trans R Soc Trop Med Hyg.

[35] World Health Organization (2000). Severe falciparum malaria. Trans R Soc Trop Med Hyg.

[36] Agusto FB, Del Valle SY, Blayneh KW, Ngonghala CN, Goncalves MJ, Li N (2013). The impact of bed-net use on malaria prevalence. J Theor Biol.

[37] Noor AM, Moloney G, Borle M, Fegan GW, Shewchuk T, Snow RW (2008). The use of mosquito nets and the prevalence of *Plasmodium falciparum* infection in rural South Central Somalia. PLoS One.

[38] Romay-Barja M, Jarrin I, Ncogo P, Nseng G, Sagrado MJ, Santana-Morales MA (2015). Rural-urban differences in household treatment-seeking behaviour for suspected malaria in children at Bata District, Equatorial Guinea. PLoS One..

[39] ICF International. Measures of malaria parasitemia prevalence in national surveys agreement between rapid diagnostic tests and microscopy. Rockville, Maryland, USA: DHS Analytical Studies No. 43; 2014. http://dhsprogram.com/publications/publication-AS43-Analytical-Studies.cfm. Accessed 10 Mar 2015.

[40] Hawkes M, Katsuva JP, Masumbuko CK (2009). Use and limitations of malaria rapid diagnostic testing by community health workers in war-torn Democratic Republic of Congo. Malar J..

